# Statistical Inference for Multi-Pathogen Systems

**DOI:** 10.1371/journal.pcbi.1002135

**Published:** 2011-08-18

**Authors:** Sourya Shrestha, Aaron A. King, Pejman Rohani

**Affiliations:** 1 Department of Ecology & Evolutionary Biology, University of Michigan, Ann Arbor, Michigan, United States of America; 2 Center for the Study of Complex Systems, University of Michigan, Ann Arbor, Michigan, United States of America; 3 Department of Mathematics, University of Michigan, Ann Arbor, Michigan, United States of America; 4 Fogarty International Center, National Institutes of Health, Bethesda, Maryland, United States of America; CNRS, France

## Abstract

There is growing interest in understanding the nature and consequences of interactions among infectious agents. Pathogen interactions can be operational at different scales, either within a co-infected host or in host populations where they co-circulate, and can be either cooperative or competitive. The detection of interactions among pathogens has typically involved the study of synchrony in the oscillations of the protagonists, but as we show here, phase association provides an unreliable dynamical fingerprint for this task. We assess the capacity of a likelihood-based inference framework to accurately detect and quantify the presence and nature of pathogen interactions on the basis of realistic amounts and kinds of simulated data. We show that when epidemiological and demographic processes are well understood, noisy time series data can contain sufficient information to allow correct inference of interactions in multi-pathogen systems. The inference power is dependent on the strength and time-course of the underlying mechanism: stronger and longer-lasting interactions are more easily and more precisely quantified. We examine the limitations of our approach to stochastic temporal variation, under-reporting, and over-aggregation of data. We propose that likelihood shows promise as a basis for detection and quantification of the effects of pathogen interactions and the determination of their (competitive or cooperative) nature on the basis of population-level time-series data.

## Author Summary

It is becoming increasingly evident that pathogens associated with infectious diseases interact amongst themselves. Pathogen interactions can occur in a co-infected host, or in host populations where they co-circulate, and they can be cooperative or competitive. Four serotypes of dengue virus, for example, can exhibit both forms of interactions – cross protection for a temporary period and followed by long-lasting enhancement. This bears important consequences for understanding the ecology and developing control and prevention measures. Detecting such interactions in a natural host population, though, can be tricky. We show that studying the phase relation of epidemic cycles, as it has been typically done, is unreliable. We assess the ability of a likelihood based method in detecting such interactions, and find that they are accurate and robust. We propose that this framework shows promise of serving as a basis for detecting and quantifying pathogen interactions.

## Introduction

Studies of infectious disease systems typically focus solely on the interaction between the host and the causative agent. This approach has served epidemiologists well, especially for antigenically stable pathogens, such as measles or chickenpox [1]–[4]. It is becoming increasingly evident, however, that a broader perspective may be necessary to take interactions among infectious agents into account [5]. The mechanisms responsible for these interactions may be either immune-mediated or ecological, and their effects may be either competitive or cooperative [6], [7].

Consider immune-mediated interactions, which are well-studied in the context of, for example, influenza virus infections in humans and other species. Exposure to a particular strain of influenza virus induces a humoral response from the host's immune system that subsequently clears the infection and is thought to confer long-lasting protection against that strain of the virus. This protective immunity may extend to other strains, depending on the similarity between the strains as measured by their “antigenic distance” [8], or the number of amino acid differences in haemagglutinin epitopes [9]. Under the resulting selective pressure, the influenza virus accumulates amino acid differences in haemagglutinin epitopes to successfully evade immunity present in the population [10], an evolutionary consequence of competition [11].

Immune-system mediated interactions may also be cooperative. An example of such an effect is the so-called *doctrine of original antigenic sin*, whereby “the antibody-forming mechanisms appear to be oriented by the initial infections of childhood so that exposures later in life to antigenically related strains result in a progressive reinforcement of the primary antibody” [12]. It is worth emphasizing that immune-mediated interactions are not necessarily restricted to genetically related pathogens. A number of recent studies speculate about the mutually beneficial interaction between HIV and malaria in Sub-Saharan Africa [13]. This interaction is thought to arise because the risk of clinical malaria is increased in HIV-1-positive individuals (due to immune-suppression) [14], [15], while malarial antigens can stimulate HIV-1 transcription and enhance replication [16], promoting HIV transmission [17]. In contrast to the competitive dynamics resulting from cross-immunity in influenza, here the interaction is facilitative.

Interactions can also arise at the population scale, driven by ecological processes. Previously, Rohani et. al. [18] proposed an interaction between measles and pertussis, two predominantly childhood diseases, via a shared susceptible pool [19]. Because infection with such acute diseases is typically followed by a period of convalescence and perhaps eventual death, an epidemic of one will lead to the temporary (due to recovery) or permanent (due to fatality) removal of susceptibles for the competing pathogen. This type of interaction –termed ecological interference– was shown to affect the phase relation between disease outbreaks [18], as well as the inter-epidemic periodicity [20].

An infectious disease system in which many of these facets are thought to be simultaneously at play is the four dengue virus serotypes [21]. After an infection, individuals are immune to subsequent homologous viruses and are thought to be protected for 2–9 months against infection with a heterologous serotype [22]. These cross-reactive antibodies, however, wane over time leading to a scenario where heterotypic viral infections are in fact enhanced through the process known as antibody-dependent enhancement (ADE) [23], [24]. Hence, with dengue, immune-mediated serotype interactions may be both competitive and cooperative, depending on the time since previous infection.

Understanding the mechanisms that drive the transmission dynamics of dengue – and other strain polymorphic pathogens – is crucial because they affect interpretation of epidemiological data [25], [26], clinical case management [27] and the design, selection and implementation of alternative control programs [26], [28]. It is thought that ADE is a major determinant of clinical pathogenesis and may explain why prior infection with a heterologous serotype is a significant risk factor for the development of the potentially fatal Dengue Hemorrhagic Fever (DHF). It is not known, however, whether ADE results in increased transmission success of dengue. Attempts to explore this question have focused on comparing the dynamics of various mathematical models with serotype-specific longitudinal data. Reported data for dengue serotypes in hyper-endemic areas such as Mexico [29], Thailand [30] and Vietnam [31] show the co-circulation of all four serotypes. A pattern of sequential serotype dominance is observed, with outbreaks of serotypes typically out of phase. This phase association has been viewed as a key dynamical signature which a variety of mathematical models of dengue have been challenged to reproduce. However, no consensus has yet emerged as to the primary mechanisms responsible for the observed oscillations in dengue serotypes. A number of studies suggest that cross-immunity may play a central role [25], [28], [32]; others have argued for ADE as the primary driving mechanism [31], [33], [34].

In an ideal world, the epidemiological impact of pathogen interactions would be quantified from individual-level infection histories observed in hosts in their natural habitats. Apart from exceptional settings [35], [36], however, such an undertaking is not feasible. It is inherently a difficult problem to scale biological processes at the level of individual organisms up to their population level consequences. Yet it is precisely the understanding of these population level implications of potential interactions that are fundamental for both public health strategies, and inferring longer-term ecological and evolutionary consequences. A step toward understanding the impact of such interactions might reside in our ability to infer their traces directly from the population level data. However, as we will show, approaches to this based on key dynamical signatures (such as phase relationships) can be unreliable as guides. Formal confrontation of mathematical models that include putative mechanisms for pathogen interactions directly with data may enable us to more effectively harvest the data's information and thus to more effectively and reliably distinguish among competing hypotheses and to quantify their relative transmission impact.

In this paper, we assess the feasibility of using a likelihood-based inference framework to detect interactions from epidemiological data. Because models with pathogen interactions can generate a rich variety of dynamics [4], [20], [28], [32], [33], [37] and may exhibit sensitivity to noise [38], it is *a priori* unclear whether such inference is feasible. The question we ask is, if several mechanisms induce dynamics that are qualitatively indistinguishable, might it nevertheless be possible to quantitatively ascertain which are most likely to be operative? Answering this question is complicated by the ubiquitous presence of stochasticity, which may very well be responsible for “patterns” that appear in data. We use a recently developed set of inference tools [39], [40] and a flexible and freely available software package [41], to formulate, estimate, and compare mechanistic models with different mechanisms of pathogen interactions. We seek to understand whether such interactions can be correctly inferred from the data. The techniques we use have been successfully applied in the context of understanding key features of cholera [42], measles [43], and malaria [44], and are amenable to testing mechanistic models of stochastic dynamics with a continuous treatment of time and noisy, incomplete observations.

In this proof-of-principle study, we show that, despite the complexity of multi-pathogen models, it is feasible to rigorously compare and distinguish among models having a realistic degree of complexity. We find that when inference is focused on pathogen interactions (*ie*, when host demography and epidemiology are known), likelihood-based inference leads generally to correct and precise conclusions. Critically, inferential power in these circumstances is determined by the strength of the underlying interaction mechanism. This conclusion is robust even where temporal dynamics are highly variable. That is, we find that accurate inference is reliably possible despite stochasticity- and initial-condition driven phase drift. We conclude that likelihood shows great promise as a basis for the detection of the effects of pathogen interactions and the determination of their (competitive or cooperative) nature on the basis of population-level time-series data.

## Methods

### Model for pathogen interactions

The model we focus upon is designed to be the simplest that admits [(i)] multiple competing interaction mechanisms, both permanent and temporary effects, and demographic stochasticity. It is a slightly simplified version of the two-pathogen compartmental model proposed by Vasco *et al.* [37]. It tracks hosts according to their pathogen-specific infection status. We bear in mind that the two pathogens in the model may represent two different strains of the same species or genetically unrelated infectious agents. In general, scaling the model up to deal with more than two interacting pathogens will be a straightforward matter, though the resulting model's complexity, *eg*, in terms of its state-space dimension, will increase geometrically with the number of interacting pathogens.

**Figure 1:**
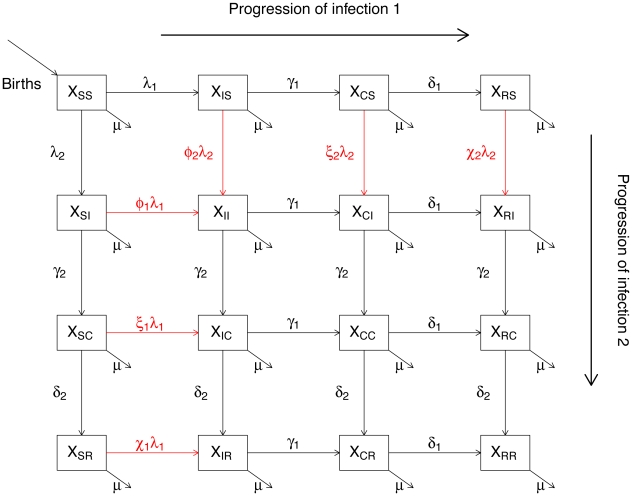
Schematics of a two pathogen model with various interaction mechanisms. Each box represents a possible host state, with individuals 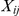 categorized according to their status with regards to the two pathogens. Letters 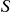, 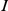, 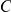, and 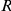 stand for susceptible, infected, convalescent, and recovered, respectively. The horizontal arrows follow the progression of a host's infection due to the first pathogen, and the vertical arrows follow the progression of the second. The diagonal arrows represent disease independent births and deaths. The transitions denoted by red arrows are affected by pathogen interaction.

As shown in Fig. 1, we assume that individuals are born susceptible to both pathogens. For each pathogen, infection dynamics follow the 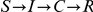 progression, where 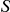, 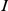 and 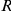 are the familiar susceptible, infectious and recovered classes, respectively. Compartment 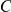 has, in previous analyses [18], [37], been used to incorporate a period of convalescence, but here might also represent, either a temporary period of immuno-suppression or strain-transcending cross-immunity or a temporary period of enhanced transmissibility associated with ADE, for example.

In this model, pathogens interact when an individual currently or previously infected with strain 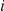 is exposed to pathogen 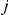. The consequence of this exposure for individuals previously exposed to strain 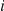 is determined by positive parameters 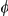, 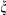 and 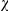, which modulate the force of infection of strain 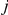, 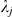 experienced by individuals in each of the 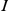, 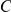, and 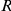 classes, respectively. Hence, if all 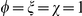, we have the null model in which the dynamics of the two pathogens are mutually independent. A value smaller than 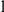 reflects either temporary (as when 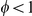 or 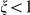) or permanent (as when 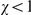) cross-immunity. Similarly, when these multipliers are greater than 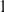, current or previous infection with one pathogen increases susceptibility to the other, either in a temporary (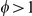 or 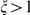) or permanent (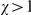) fashion. This model assumes that all pathogen interactions are via modulation of host susceptibility. In reality, interactions may also operate via transmissibility. Here we ignore effects of heterotypic infections on transmissibility, as explored by, for example, [28].

The model also accounts for host demography in that births replenish the susceptible pool, and natural deaths remove hosts from each compartment. These rates are assumed independent of disease status and are both fixed at 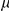. Thus the host population size is held constant.

#### Deterministic skeleton of the model

In a deterministic setting, the model is described by 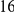 ordinary differential equations. Equations for each state can be read directly from Fig. 1. In particular, each arrow is associated with an instantaneous flux which is the product of a *per capita* rate and the number of individuals in the source box. The *per capita* rates are symbolized using Greek letters. The equations of state for any compartment are obtained by summing the fluxes associated with all arrows pointing into that compartment and subtracting the sum of the fluxes associated with arrows pointing out of it. For example, if we let 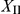 denote the number of individuals currently infected with both strains, then
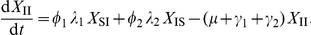
The entire set of differential equations are presented in the supplementary information (Text S1).

The only non-linearities in the model arise as usual via the frequency-dependent transmission process. In particular, the respective forces of infection terms for the two pathogens are given by 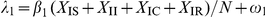 and 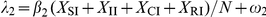. Here, 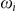
s are immigration terms, which are constant through time.

#### Stochastic model

To model the presence of stochasticity, we translate the ODE defined above into a stochastic process model. We do this by considering each flux between compartments to be a random process. In particular, we assume that, over a small time interval of duration 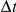, the *per capita* rates are constant and that the fluxes out of each compartment are independent, multinomial random variables. Thus for example, if we focus on the 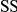 compartment, there are three ways of exiting: infection by agent 1, infection by agent 2, and death. By assumption, the *per capita* probability of exit in the interval 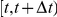 is constant and, letting 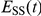 denote the number that actually do exit 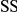 in this interval, we have 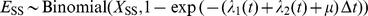. Among those that exit, the numbers of hosts respectively infected with agent 1, infected with agent 2, and dying over this time interval are distributed as 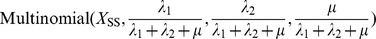. Finally, to model extra-demographic stochasticity, we include a gamma-distributed multiplicative white noise, 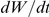, in the transmission process [43], [45].

In full, the pathogen-specific forces of infection are given by:
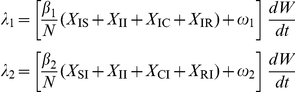



### Inference technology

To infer the nature of pathogen interactions in systems with variability in phase relationships, we utilize the framework of partially observed Markov processes [41]–[43]. This consists of three major components: (i) *the data*; (ii) *the ‘process’ model*, proposed to describe the underlying epidemiological and demographic processes (described in section “Stochastic Model”); and finally (iii) *the observation model*, proposed to describe the process by which the data are generated and linking the process model with the reported data.

We assume the data consist of monthly pathogen/serotype-specific case notifications. Since we consider two pathogens, the data comprise parallel time series data 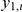 and 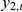 (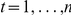) are related to the true number of infections via a Poisson distribution. Specifically, if 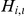 is the total number of new recoveries in month 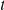 for pathogen 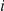, and 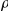 is the reporting probability, then monthly case notifications 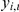 is assumed to have been drawn from a Poisson distribution with mean 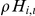. The data sets we use to challenge our inference technique are realizations of this model. We use 40 years of simulated data, unless otherwise stated.

For each simulated data set, we compute profile log-likelihoods over the parameters of interest, 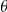. The log-likelihood function may be expressed 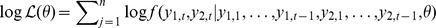, *ie*, as the sum of conditional log-likelihoods of 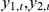 given 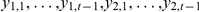 and parameters 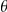. These quantities are computed using a Sequential Monte Carlo (SMC) algorithm [40], [46]. Each SMC calculation uses 30,000 particles. To estimate Monte Carlo error, we repeat each SMC calculation 5 times at each parameter. The number of SMC particles and the resolution of the grid over 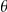 are the only algorithmic parameters: it would be straightforward to further reduce Monte Carlo noise in our estimates by using more particles in the SMC calculations and/or a finer grid over 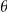. The cost of doing so is purely the greater computational effort required. For further details, refer to the supplementary information (Text S1).

## Results

We present our results in several parts. First, we devote subsections titled “System dynamics” and “Unreliability of phase as an indicator of interactions” to an examination of the phase association between strains under a variety of interaction scenarios. In subsection “Basic identifiability of pathogen interaction mechanisms”, we report our findings from the inference study. Subsequently (subsection titled “Stress testing the inference approach”), we examine the robustness of our results to a number of realistic complications.

### System dynamics

In the absence of pathogen interaction, the dynamics of our unforced deterministic system are characterized by damped oscillations. However, interaction between pathogens can lead to sustained oscillations depending on parameter values [4], [37]. When oscillations exist, cooperative interactions tend to generate in-phase cycles while competitive interactions tend to lead to out-of-phase oscillations. However, as Kamo & Sasaki [38] showed in a somewhat similar, but seasonally forced, system, the phase relationship between strains can be sensitive to stochasticity. Specifically, they demonstrated that noise can destabilize the in-phase solution, leading to asynchronous fluctuations. Similarly, in our stochastic system, phase relationships are variable. For all parameter values we examined, stochastic trajectories drift in and out of phase.

### Unreliability of phase as an indicator of interactions

**Figure 2:**
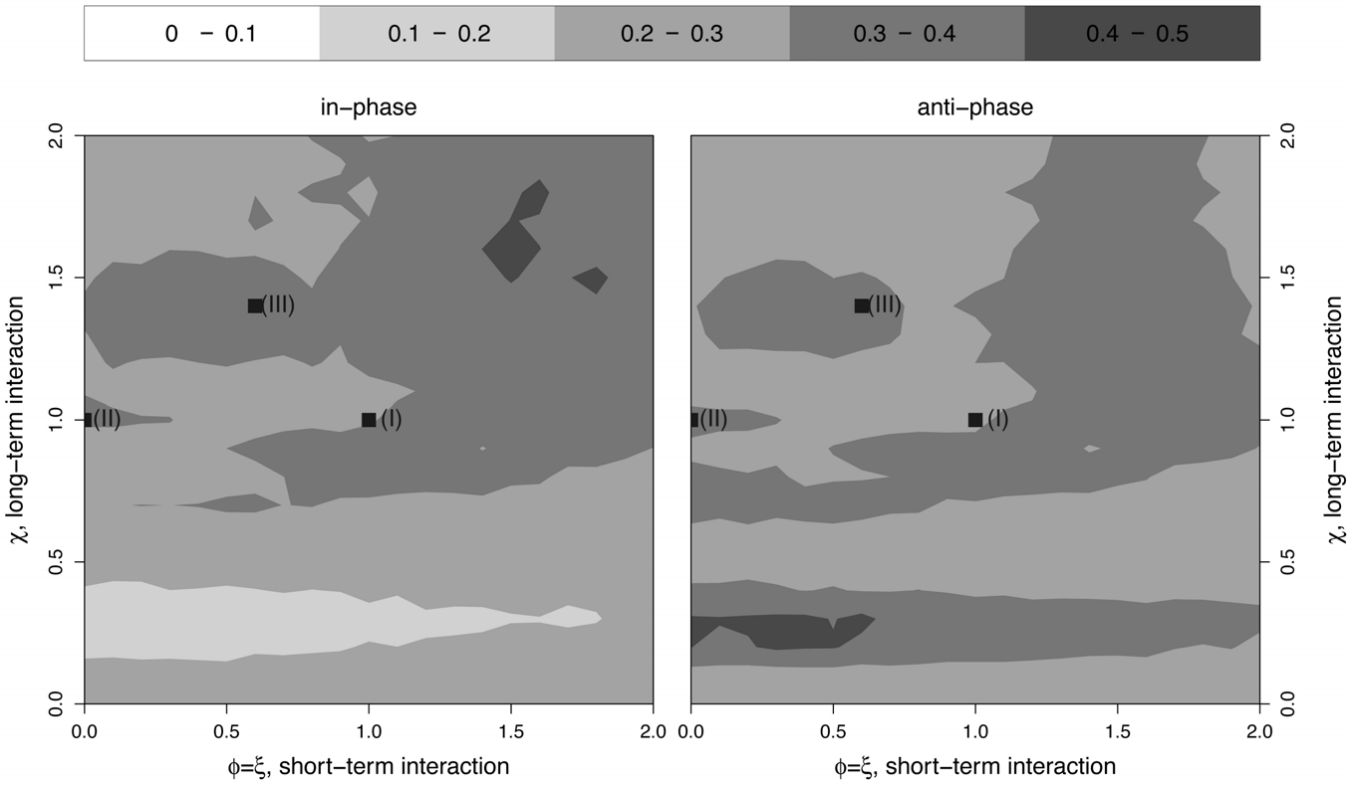
Phase relation between the two epidemics in the simulation of the model. Level contours plot the fraction of time epidemics are in-phase [Left], and anti-phase [Right]. Phase difference is calculated by considering 5000 years of simulation (100 years of transients are excluded), computing the fraction of the time series during which strains are in-phase and anti-phase and averaging these fractions over 40 stochastic replicates. Strains are categorized as in phase if the phase difference is less than an eighth of the period, and anti-phase if the difference is 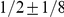 of a period. The three points marked (I), (II) and (III) are distinct scenarios examined in our inference tests. Model parameters are as in Table 1, with 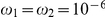.

To assess the reliability of between-strain phase relationship as an indicator of the nature of pathogen interactions, we performed a simulation study. We varied the interaction parameters (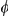, 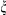, 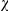) across broad ranges, simulating 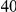 realizations of 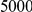 yr duration at each point in parameter space. For each combination of parameters, the phase difference in strain-specific incidence generally varies with time. Fig. 2 shows the fraction of time during which oscillations are in-phase (left) and anti-phase (right), as a function of the strength and sense (cooperative versus competitive) of both short- and long-term interaction. Even in multiply replicated time series of 5000 yr duration, no consistent association between the cooperative or competitive nature of epidemiological interactions and phase relationship emerges. While permanent cross-immunity (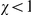), for example, frequently leads to out-of-phase dynamics, it is also associated with in-phase solutions 10%–20% of the time. More generally, for any combination of parameters, there is a moderate chance (between 10% to 50%) of observing either in-phase or anti-phase trajectories. The important practical implication is that phase relationship may be a poor predictor of the mechanism of pathogen interaction. Indeed, phase relationship alone appears to be of little use in indicating even the cooperative or competitive sense of pathogen interactions.

### Basic identifiability of pathogen interaction mechanisms

To establish whether likelihood offers an improved basis for inferring the nature of pathogen interactions from epidemiological data, we performed another simulation study. We focused on parameters intended to be typical of closely related pathogen strains. In particular, we assumed symmetry between interacting pathogens, *ie*, 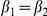, 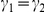, 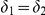, 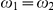, 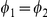, 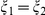, and 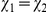. To keep the complexity manageable in this proof-of-principle study, we focused on the interactions parameters by assuming the strictly epidemiological parameters (contact rates, infectious periods, immigration rates, and durations of the temporary 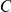 stage) to be known. Moreover, we assumed the short-term interaction parameters to be identical, *ie*, 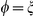. Table 1 gives the values to which these parameters were set. With these parameters, the net reproductive number, 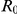, is 2.7 (in the absence of pathogen interaction).

**Table 1:** Model parameters and their corresponding ranges.

Parameter	Description	Range
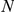	Host population size	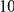 million
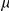	*Per capita* host birth/mortality rate	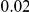 per year
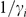	Average infectious period	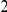 weeks
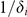	Average convalescent period	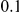 years
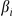	Transmission rate	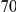 per year
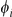	Interaction during infectious period	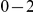
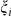	Interaction during convalescent period	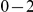
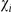	Interaction during recovered period	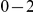
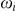	Force of infection due to immigration	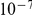
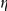	Std. deviation of the gamma-distributed white noise ( 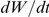 )	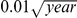
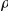	Reporting rate	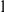

We examined the identifiability of the interaction parameters in three distinct scenarios (Fig. 2):

Scenario I: No pathogen interactions, 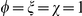. Since each pathogen is independent of the other, this serves as a null model.

Scenario II: Perfect short-term cross-protection, no long-term interaction, 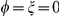, 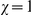. The ecological interference proposed to explain measles-pertussis interactions (eg, [18]) is an example.

Scenario III: Moderate short-term cross-protection, permanent enhancement, 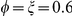, 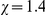. These effects have been posited for the 4 dengue serotypes in hyper-endemic regions.

For each scenario, we present log-likelihood profiles for the two interaction parameters of interest: short-term (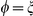) and long-term (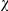). We plot differences of log-likelihoods, 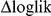, and compute confidence regions using likelihood ratio tests. We scale log-likelihoods such that the 95% confidence region corresponds to 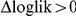. Further details of the profile likelihood construction are provided in the supplementary information (Text S1).

#### Scenario I: No interaction

**Figure 3:**
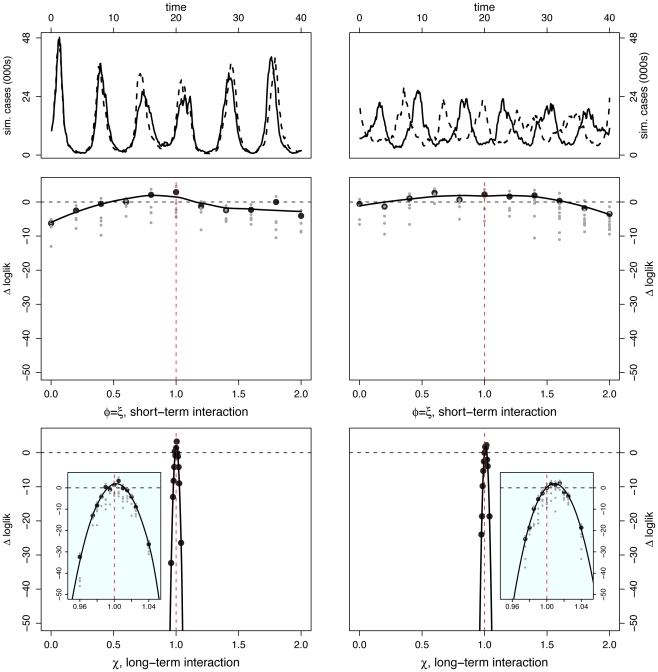
Inference under scenario I: No pathogen interaction. Inference is carried out for two separate data sets constructed from the same set of parameter values – results are shown in [Left] and [Right] columns for each data set. [Top] Simulated case-data for the two infections are plotted in solid and dashed lines. Log-likelihood profiles for parameters describing the short (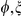) [Middle] and the long term (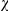) [Bottom] interactions. In the insets, we show close-ups of the profiles near the peaks. Plotted 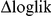 are relative difference in the raw log-likelihood from the reference point set at 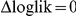, indicated by the horizontal dashed line. 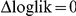 represents the 95% confidence interval–parameter values corresponding to a positive 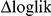 are within the confidence bound. The gray dots indicate the repeated likelihood estimates (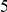 replicate SMC calculations for each profile point, 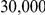 particles in each SMC calculation). The profiles are created by fitting a smooth line through the log of the arithmetic mean likelihoods (shown in black dots). The vertical red dashed line is plotted at the actual parameter value used to generate the simulated case-data. Parameters not shown in the graph are taken from Table 1.

Here, the pathogens are independent; oscillations are noisy and phase relationship is variable. In particular, when epidemics are observed over only 40 yr, pathogen-specific oscillations can appear to be in phase, out of phase, or neither. We selected two superficially different data sets (Fig. 3): one realization displays large-amplitude oscillations with strongly in-phase dynamics; the other, smaller-amplitude fluctuations and strongly asynchronous dynamics.

Perhaps surprisingly, the log-likelihood profiles derived from these two data sets are similar for both short- and long-term interaction parameters. Despite the noticeable qualitative dynamical differences between these data sets, the long-term interaction parameter, 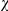, is extremely well identified, with a narrow confidence interval embracing the true value. The short-term interaction parameter (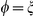) is less well identified, as can be seen from the flatter likelihood profile. That these parameters are estimated correctly if imprecisely is indicated by the fact that the true value is contained within the confidence interval.

#### Scenario II: Strictly temporary cross-immunity

**Figure 4:**
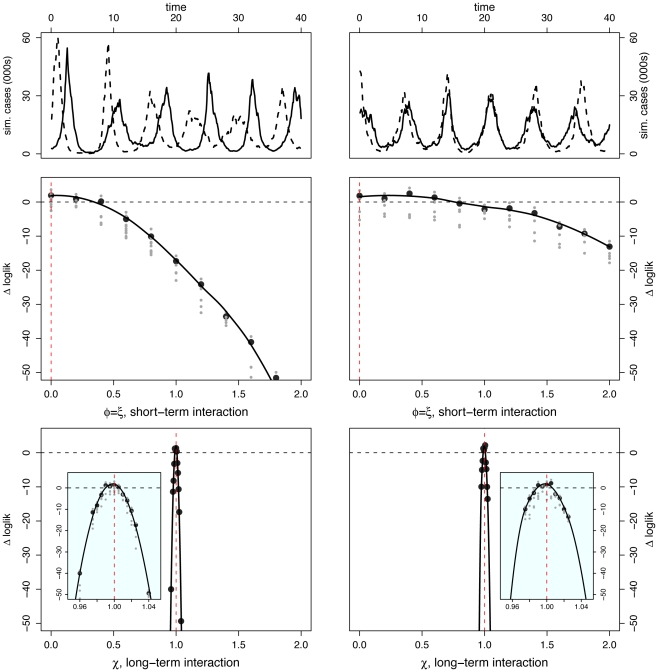
Inference under scenario II: Temporary cross-immunity. Inference is carried out for two separate data sets constructed from the same set of parameter values – results are shown in [Left] and [Right] columns for each data set. [Top] Simulated case-data for the two infections are plotted in solid and dashed lines. Log-likelihood profiles for parameters describing the short (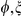) [Middle] and the long term (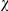) [Bottom] interactions. In the insets, we show close-ups of the profiles near the peaks. Plotted 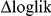 are relative difference in the raw log-likelihood from the reference point set at 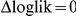, indicated by the horizontal dashed line. 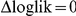 represents the 95% confidence interval – parameter values corresponding to a positive 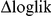 are within the confidence bound. The gray dots indicate the repeated likelihood estimates (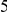 replicate SMC calculations for each profile point, 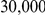 particles in each SMC calculation). The profiles are created by fitting a smooth line through the log of the arithmetic mean likelihoods (shown in black dots). The vertical red dashed line is plotted at the actual parameter value used to generate the simulated case-data. Parameters not shown in the graph are taken from Table 1.

In this scenario, we set 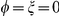, representing a strong competitive interaction ( *ie*, perfect cross-immunity or complete convalescent isolation) during the infectious and convalescent periods (a total of 7 wk). We set 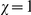, denoting no long-term interaction. In Fig. 4, we present time series for two independent model realizations. Again, in one, oscillations appear to be out of phase and in the other, in phase. As before, we find likelihood yields accurate estimates, with greater precision in the case of the long-term interaction parameter. Log-likelihood profiles indicate that both the temporary, perfect cross-immunity (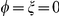) and the absence of long term interaction (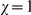) are quite well identified.

#### Scenario III: Temporary, partial cross-immunity and long-term enhancement

**Figure 5:**
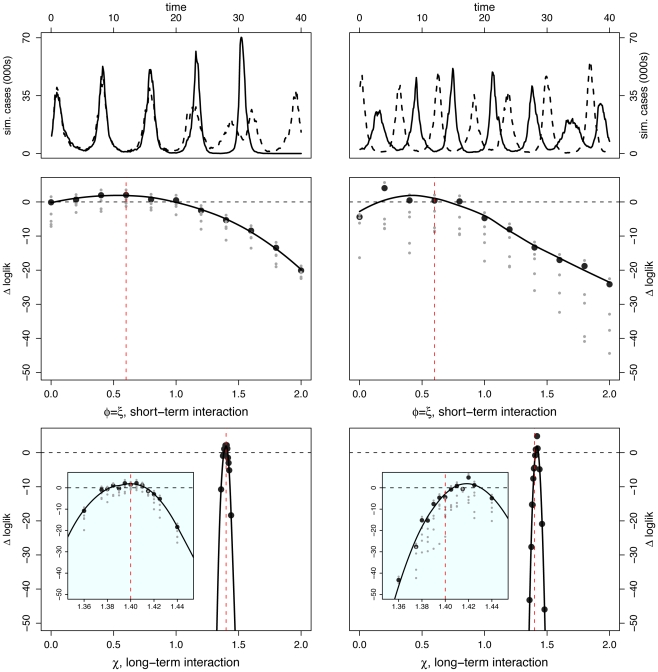
Inference under scenario III: Partial and temporary cross-immunity, and delayed but permanent enhancement. Inference is carried out for two separate data sets constructed from the same set of parameter values – results are shown in [Left] and [Right] columns for each data set. [Top] Simulated case-data for the two infections are plotted in solid and dashed lines. Log-likelihood profiles for parameters describing the short (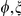) [Middle] and the long term (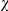) [Bottom] interactions. In the insets, we show close-ups of the profiles near the peaks. Plotted 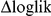 are relative difference in the raw log-likelihood from the reference point set at 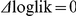, indicated by the horizontal dashed line. 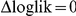 represents the 95% confidence interval – parameter values corresponding to a positive 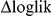 are within the confidence bound. The gray dots indicate the repeated likelihood estimates (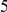 replicate SMC calculations for each profile point, 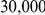 particles in each SMC calculation). The profiles are created by fitting a smooth line through the log of the arithmetic mean likelihoods (shown in black dots). The vertical red dashed line is plotted at the actual parameter value used to generate the simulated case-data. Parameters not shown in the graph are taken from Table 1.

In the third scenario, we consider both permanent and temporary immunological interactions. During the infectious and convalescent phases, hosts are assumed partially immune to secondary infection (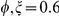). After recovery, host susceptibility to the other pathogen is enhanced (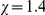). As we have seen before, phase relationship is variable and both in- and out-of-phase dynamics of 40 yr duration are possible (Fig. 2). Among typical realizations, the degree of asynchrony evident in the top right panel of Fig. 5 is relatively but not extremely unusual; we comment more on this below.

Again, the likelihood profiles establish that both long- and short-term interaction parameters are well identified, the long-term parameters with higher precision. That the true parameter value lies outside the 95% confidence interval for the anti-phase time series reflects the fact that the simulated data set is somewhat atypical. Even in this case, however, the null hypothesis (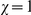) can be rejected, with the estimate within 2% of the true value. It is interesting that the degree of enhancement is over-estimated in this case despite the strongly anti-phase nature of the dynamics.

### Stress testing the inference approach

Having established that likelihood-based inference is computationally feasible in this system and can yield accurate estimates of interaction parameters, we now push further in search of the approach's limitations.

#### Trade-off between the estimated strength and duration of interaction

**Figure 6:**
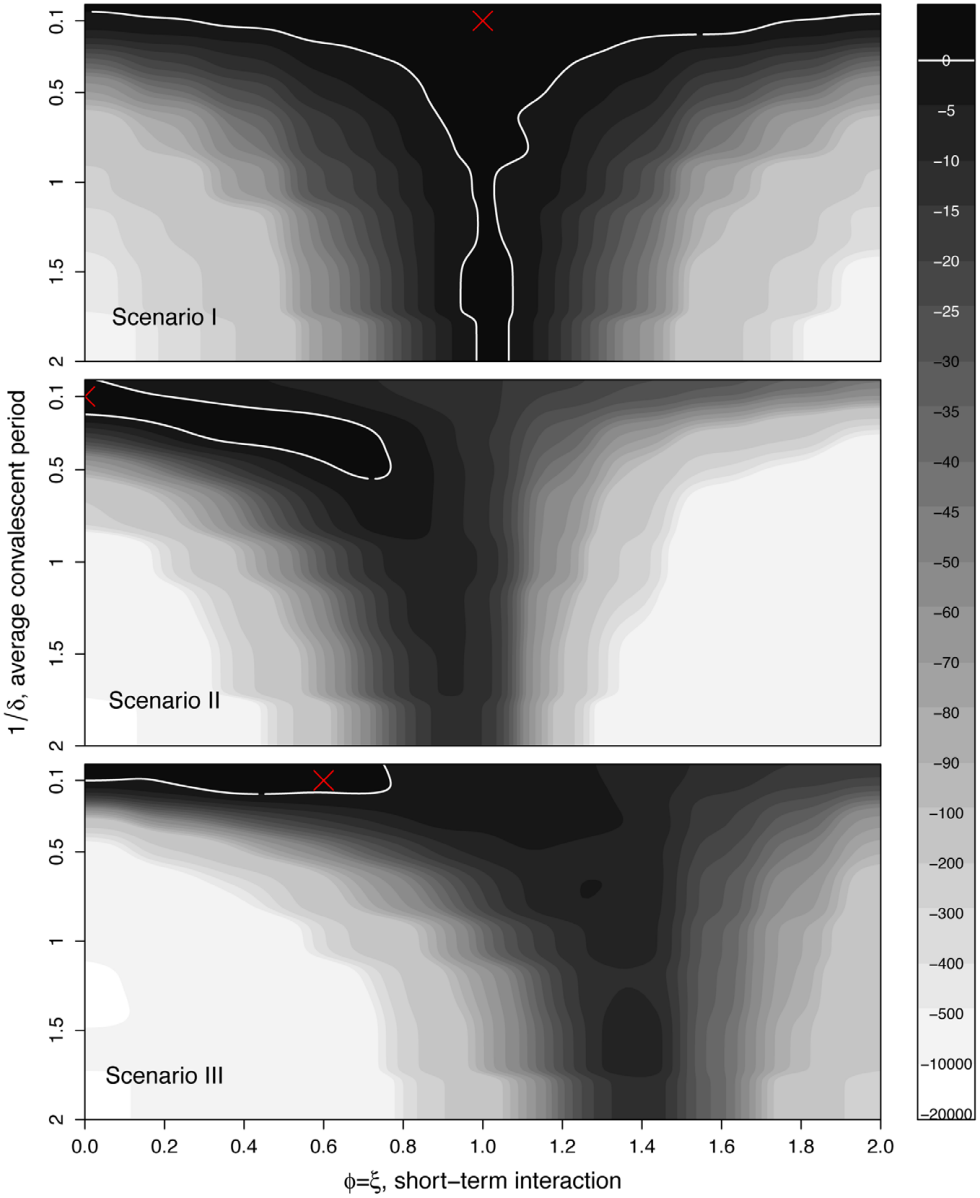
Two-parameter profile log-likelihood surfaces: strength and duration of the short term interaction. 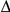
loglik contours (strength of the short term interaction, 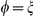, on the horizontal axis and the average duration of such interaction, 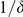, on the vertical axis) for (top to bottom) scenarios I, II, and III. The red cross indicates the actual parameter values. Darker contours correspond to parameter regions that have higher log-likelihood, and more consistent with the data. Solid white lines show the 95% confidence regions. Parameters as in Table 1.

The dynamical consequences of an temporary pathogen interaction depend not only on its strength but also on its duration [18], [37]. From an inference perspective, strength and duration may trade off against one another: increasing duration may masquerade as increased strength and vice-versa. In the preceding section, we assumed the mean duration of the short term interaction (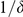) to be known. We seek now to understand whether this parameter can be identified simultaneously with the interaction strengths. Fig. 6 shows how the likelihood depends on short-term interaction strength and duration in each of the three scenarios described above. In the absence of all interaction (scenario I, top panel), there is a noticeable likelihood ridge spanning the full range of 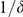 at 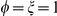. This indicates that the duration of the (non-existent) interaction is, not surprisingly, unidentifiable. One also notes that the ridge broadens as the 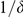 decreases. Thus the fact that the data contain little evidence for the pathogen interaction can be interpreted either as an interaction of zero strength but arbitrary duration or a very short interaction of arbitrary strength.

In scenario II (temporary, perfect cross-immunity), the likelihood profile reveals that, although the trade-off described above is evident, the interaction duration is quite well identified. Importantly, the null case (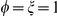) is well outside the 95% confidence region. In scenario III (temporary, partial cross-immunity and long-term enhancement), the duration of the temporary phase is very well identified. This is due in part to the fact that under this scenario, the temporary and long-term interactions are of opposite senses.

#### Length of time series

**Figure 7:**
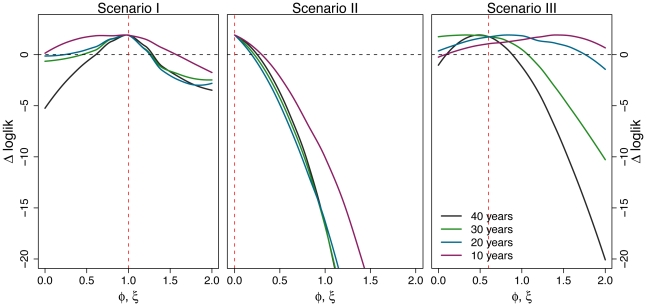
Inference precision and accuracy as a function of time series length. We compare the shape of the log-likelihood profiles for short term interaction 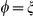 as the size of the data varies, in three different scenarios. Other parameters are taken from Table 1.

With 40 yr of monthly data, likelihood appears a promising basis for inferring pathogen interactions. In Fig. 7, we quantify how the quality of inference degrades as the time series become shorter. As should be expected, the likelihood profiles are flatter ( *ie*, estimates are less precise) with less information. It remains relatively easy to distinguish the lack of interaction (scenario I), as well as the presence of strong negative interaction when this is the sole interaction (scenario II). In scenario III, however, monthly data sets of less than 40 yr contain insufficient information to reject the null hypothesis for the short-term interaction (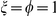).

#### Under-reporting bias

So far, we have assumed that our case-report data are an unbiased reflection of the true number of infections, i.e. reporting rate, 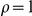. Next, we assess to what extent our ability to infer the sense and strength of interactions is hampered by systematic under-reporting. We construct the log-likelihood profiles, shown in Fig. S2 in the supplementary information (Text S1), using the same data set as in Fig. 5[Right], but assuming that, on average, only 50% of cases are reported (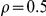). The figure shows that the log-likelihood profiles remain essentially unchanged. Hence, in the presence of known under-reporting, little information is lost, and minimal bias is introduced.

**Figure 8:**
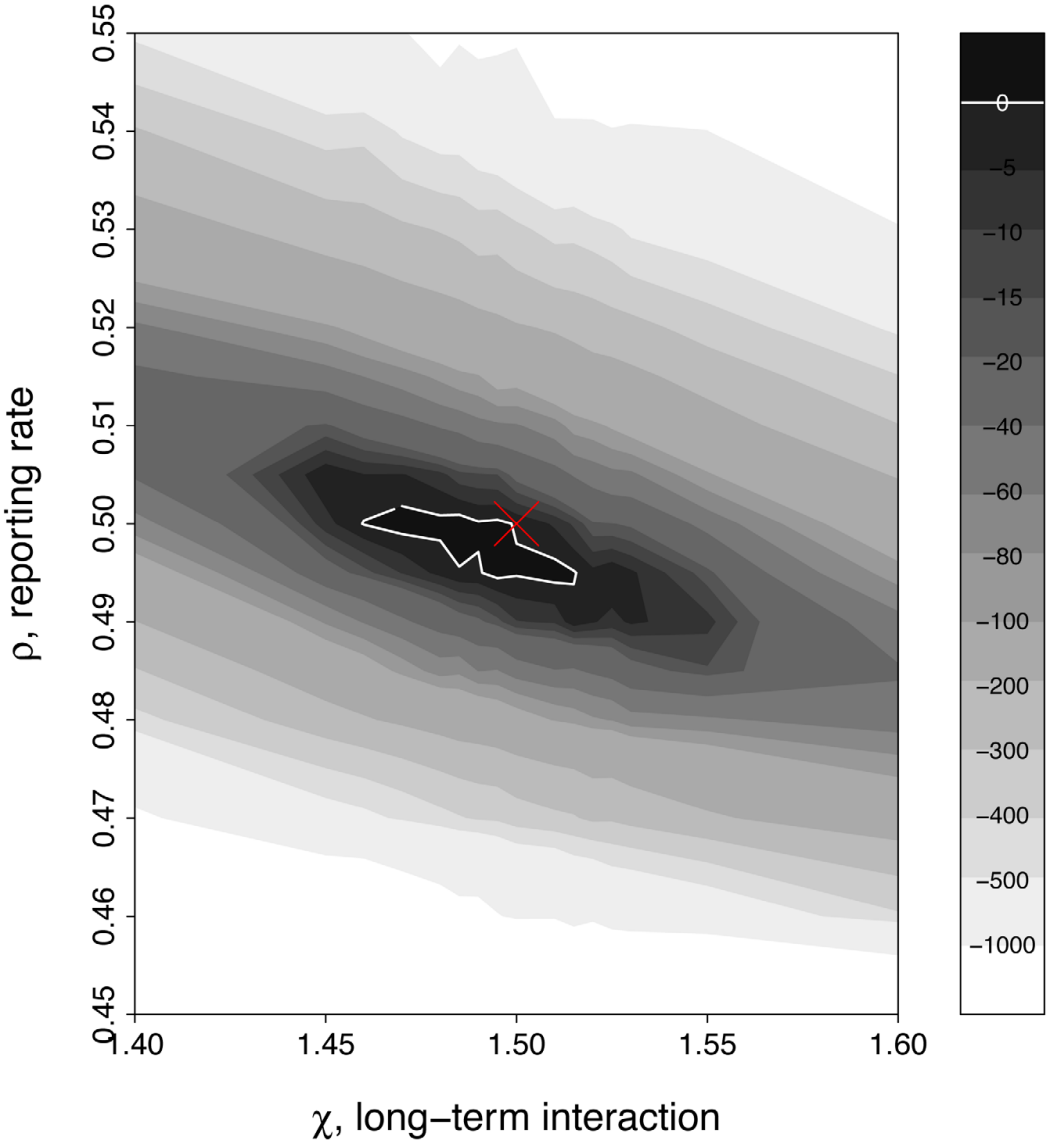
Simultaneous inference of under-reporting and long-term interaction. Plotted are 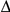
loglik contours (strength of the long-term interaction, 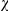, on the horizontal axis and the reporting rate, 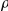, on the vertical axis). Marked in red crosses are the actual parameter values for 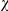 and 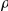. Darker contours correspond to parameter regions that have higher log-likelihood. Solid white lines show the 95% confidence regions. For this figure, short term interaction 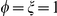. Parameters not shown in the graph are taken from Table 1. See Fig. S3 in the supplementary information (Text S1) for the simulated data and corresponding profiles.

A more rigorous challenge arises when reporting bias is not known *a priori* and must be inferred along with interaction parameters. We take one such scenario in which both reporting rate, 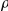, and long-term interaction, 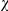, are unknown. The log-likelihood profiles (Fig. 8) show that these parameters can be identified simultaneously.

#### Aggregated data

**Figure 9:**
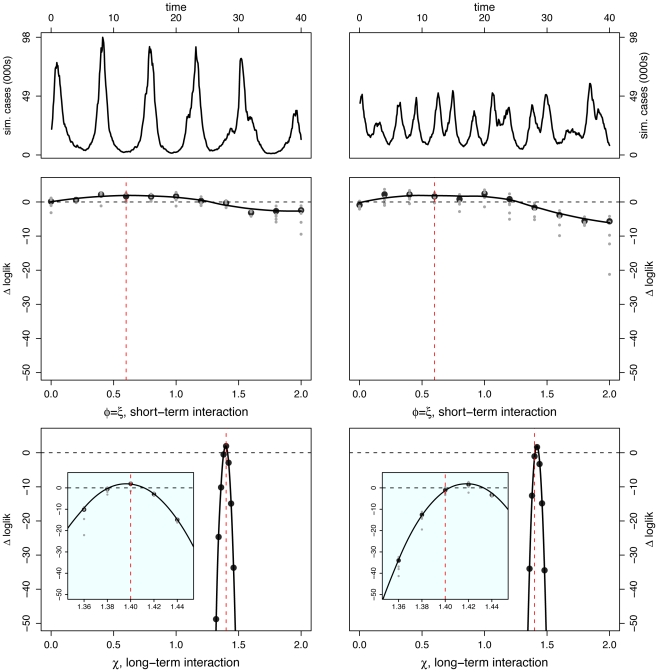
Inference under scenario III with aggregated data. Inference is carried out for two separate data sets constructed from the same set of parameter values – results are shown in [Left] and [Right] columns for each data set. [Top] These are the same data sets used to make Fig. 5. For each data set, the two time series are added together to form a single aggregated time series. Log-likelihood profiles for parameters describing the short (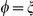) [Middle] and the long term (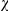) [Bottom] interactions. In the insets, we show close-ups of the profiles near the peaks. Plotted 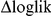 are relative difference in the raw log-likelihood from the reference point set at 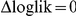, indicated by the horizontal dashed line. 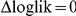 represents the 95% confidence interval – parameter values corresponding to a positive 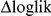 are within the confidence bound. The gray dots indicate the repeated likelihood estimates (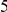 replicate SMC calculations for each profile point, 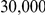 particles in each SMC calculation). The profiles are created by fitting a smooth line through the log of the arithmetic mean likelihoods (shown in black dots). The vertical red dashed line is plotted at the actual parameter value used to generate the simulated case-data. Parameters not shown in the graph are taken from Table 1.

We have assumed in the foregoing that the data are strain specific, *ie*, that accurate strain typing is possible. It is frequently the case that strain identification is unavailable, impossible, or ambiguous. In the extreme case, incidence data represent an aggregate across strains. We now ask whether with such aggregated data it is possible to infer the strength and sense of strain interactions. To address this, we sum the strain-specific incidence data from Scenario III (Fig. 5,[Top]). The resulting log-likelihood profiles are shown in Fig. 9. The broader confidence intervals reflect the fact that information has been lost by aggregation (*cf* Fig. 5). Yet, the longer term interaction is still well identified, irrespective of phase relation.

#### Unknown initial conditions

In our proof-of-principle study thus far, we have assumed accurate knowledge of initial conditions–the fraction of the host population in each compartment immediately before the first observation. In a real world application, these would need to be estimated along with the other parameters. In Fig. S4 in Text S1, for the data sets in Fig. 5, we construct profile likelihoods as before but conditional on the estimated initial conditions. That is to say, for each value of the interaction parameters, we first estimate the initial conditions (see the Text S1 for implementation details) and compute likelihoods based on these values. Results show that while uncertainty regarding initial conditions reduces the precision of interaction parameter estimates, the fundamental identifiability is unaffected.

## Discussion

In recent decades, much work has focused on identifying the immunological consequences of infection with one pathogen for subsequent infection with other co-circulating pathogens. This is most obviously applicable to strain-polymorphic pathogens, such as influenza and dengue viruses, but is increasingly thought to affect unrelated infections, including the mutual enhancement of HIV and malaria [13], TB and macroparasitic infections [47], competitive/mutualistic dynamics of intestinal worms [35], [48], and an entire community of parasites co-circulating in wildlife populations [36]. There are two practical problems. First, it is imperative to establish the extent to which processes of enhancement or exclusion occurring at the level of an individual impact large scale transmission dynamics. Second, in instances where multiple competing mechanisms for pathogen interaction have been mooted, it is important to know whether analyses of incidence data can facilitate hypothesis testing.

We have approached these problems by attempting to infer potential interactions from simulated case-notification data. Our choice of a two-pathogen model is deliberately simple. In general, concurrent or prior infection with heterotypic pathogens may modify host susceptibility, transmissibility, virulence, and infection duration, with concomitant impacts on epidemiology. Here, we focus strictly on interactions that affect host susceptibility. Our transmission model is sufficiently flexible and likelihood sufficiently powerful as a basis for inference that investigations of other mechanisms can be straightforwardly accommodated.

We are encouraged to find that, in the optimistic case where the epidemiological traits of each pathogen (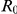 and infectious period) and seroepidemiology (initial conditions) are known, it is clearly possible to correctly infer the strength and nature of interactions from longitudinal data. Moreover, even when initial conditions are not known, it is possible to estimate them, with little loss of precision. Examining three distinct scenarios, we have described accurate inference of the presence/absence of an interaction, its strength and, promisingly, the confident identification of multiple co-occurring modes of interactions. Not surprisingly, our ability to infer an interaction is determined by its dynamical impact, with permanent effects better identified than interactions that operate over short time scales.

It is important to point out that much of the work on serotype dynamics of dengue [28], [32], [33] or interference effects between measles and pertussis [18], [19] has focused on phase association as a key dynamical signature. In our stochastic unforced model, we find that phase relation is highly variable (Fig. 2) and is, by itself, an unreliable indicator of the type and the intensity of pathogen interaction. To quantify the impact of phase structure in our inference, for each scenario, we deliberately picked two data sets that showed qualitatively different relative phases. Somewhat surprisingly, our ability to make inference about underlying interactions is not driven by the correlation dynamics of the data. This suggests that phase relation–visually suggestive though it may be–is not a characteristic feature that provides reliable information about pathogen interaction.

We need to place the relatively encouraging results of this proof-of-principle study within the context of our central–and at times rather optimistic–assumptions. As we have shown, the length of the time-series data directly affects the power of the inference. Within the confines of this project, we find that 40 yr of monthly data appear sufficient for robust inference. However, restricting the data to 20 yr substantially weakens the inference, particularly concerning short-term interactions.

Relaxing the assumption of 100% reporting fidelity is an important reality check. We find that under-notification does not significantly impact the identifiability of key model parameters, assuming that the reporting precision is known. Even when the extent of under-reporting is not known and must be estimated along with other parameters, however, our results indicate that pathogen interactions remain identifiable (see Figs. S3 in Text S1, & Fig. 8). Another possible data limitation is aggregation, particularly when for disease systems with multiple genetically related strains or serotypes. Our explorations of this problem suggest, although the information on the short term interaction is diluted, it need not substantially impair the correct identification of the interaction.

The inference tests carried out here assumed a relatively low basic reproduction ratio (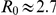), a short mean duration of temporary interaction (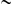
7 weeks) in a large population (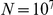). The power of these inferential tests is likely to change with disease epidemiology and population demographics. As we show in Fig. S1 in Text S1, interactions are more clearly identifiable with a higher 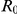. This is to be expected since more interactions occur when infections are more contagious. Similarly, we expect temporary interactions to be more clearly identifiable when they operate over longer periods. In contrast, strain interactions in smaller populations–with their noisier dynamics and lower-amplitude outbreaks–should be less precisely identifiable.

All of our inference tests have relied on an important assumption–perfect knowledge of disease epidemiology. In any real world application of our methodology, the transmission rate, infectious period and initial conditions will likely need to be estimated alongside the interaction parameters. It remains to be seen whether likelihood-based inference is up to such a challenge.

## Supporting Information

Text S1 In the supplementary information, we provide the following: 1. The complete deterministic skeleton of the model in Table S1. 2. A brief description of the inference technology used in the paper. 3. Details on the construction of log-likelihood profiles and surfaces. 4. An example examining the effect of changing epidemiology, with Fig. S1. 5. Supplementary information on under-reporting, with Fig. S2, and Fig. S3. 6. An example where initial conditions are estimated along with the interaction, with Fig. S4.
(PDF)
