# Supplementary material for: Statistical Inference for Multi-Pathogen Systems
Source: PLoS Comput Biol. 2011 Aug 18;7(8):e1002135. doi: 10.1371/journal.pcbi.1002135 (PMC3158042; doi:10.1371/journal.pcbi.1002135)
Supplement: Text S1 — In the supplementary information, we provide the following: 1. The complete deterministic skeleton of the model in Table S1. 2. A brief description of the inference technology used in the paper. 3. Details on the construction of log-likelihood profiles and surfaces. 4. An example examining the effect of changing epidemiology, with Fig. S1. 5. Supplementary information on under-reporting, with Fig. S2, and Fig. S3. 6. An example where initial conditions are estimated along with the interaction, with Fig. S4. (PDF) [file pcbi.1002135.s001.pdf]

Text S1

Sourya Shrestha[1,2,*], Aaron A. King[1,3,4], Pejman Rohani[1,2,4]
1 Department of Ecology & Evolutionary Biology, University of Michigan, Ann Arbor, MI 48109, USA
2 Center for the Study of Complex Systems, University of Michigan, Ann Arbor, MI 48109, USA
3 Department of Mathematics, University of Michigan, Ann Arbor, MI 48109, USA
4 Fogarty International Center, National Institutes of Health, Bethesda, MD 20892, USA
∗ E-mail: Corresponding sourya@umich.edu

Deterministic skeleton of the model

The deterministic skeleton of the model is completely described by the 16 equations given in Table S1:

Table S1. Deterministic skeleton of the model. The equations are organized in a grid corresponding to the schematic layout of the compartments shown in Fig. 1. The symbols are described in Table 1.

$\frac{dX_{SS}}{dt} = \mu N - (\lambda_1 + \lambda_2 + \mu)X_{SS}$	$\frac{dX_{IS}}{dt} = \lambda_1 X_{SS} - (\gamma_1 + \phi_2\lambda_2 + \mu)X_{IS}$	$\frac{dX_{CS}}{dt} = \gamma_1 X_{IS} - (\delta_1 + \xi_2\lambda_2 + \mu)X_{CS}$	$\frac{dX_{RS}}{dt} = \delta_1 X_{CS} - (\chi_2\lambda_2 + \mu)X_{RS}$
$\frac{dX_{SI}}{dt} = \lambda_2 X_{SS} - (\phi_1\lambda_1 + \gamma_2 + \mu)X_{SI}$	$\frac{dX_{II}}{dt} = \phi_1\lambda_1 X_{SI} + \phi_2\lambda_2 X_{IS} - (\gamma_1 + \gamma_2 + \mu)X_{II}$	$\frac{dX_{CI}}{dt} = \xi_2\lambda_2 X_{CS} + \gamma_1 X_{II} - (\delta_1 + \gamma_2 + \mu)X_{CI}$	$\frac{dX_{RI}}{dt} = \delta_1 X_{CI} + \chi_2\lambda_2 RS - (\gamma_2 + \mu)X_{RS}$
$\frac{dX_{SC}}{dt} = \gamma_2 X_{SI} - (\xi_1\lambda_1 + \delta_2 + \mu)X_{SC}$	$\frac{dX_{IC}}{dt} = \xi_1\lambda_1 X_{SC} + \gamma_2 X_{II} - (\gamma_1 + \delta_2 + \mu)X_{IC}$	$\frac{dX_{CC}}{dt} = \gamma_1 X_{IC} + \gamma_2 X_{CI} - (\delta_1 + \delta_2 + \mu)X_{CC}$	$\frac{dX_{RC}}{dt} = \delta_1 X_{CC} + \gamma_2 X_{RI} - (\delta_2 + \mu)X_{RC}$
$\frac{dX_{SR}}{dt} = \delta_2 X_{SC} - (\xi_1\lambda_1 + \mu)X_{SR}$	$\frac{dX_{IR}}{dt} = \chi_1\lambda_1 X_{SR} + \delta_2 X_{IC} - (\gamma_1 + \mu)X_{IR}$	$\frac{dX_{CR}}{dt} = \gamma_1 X_{IR} + \delta_2 X_{CC} - (\delta_1 + \mu)X_{CR}$	$\frac{dX_{RR}}{dt} = \delta_1 X_{CR} + \delta_2 X_{RC} - \mu X_{RR}$

The two forces of infection are: $\lambda_1 = \frac{\beta_1}{N}(X_{IS} + X_{II} + X_{IC} + X_{IR}) + \omega_1$ and, $\lambda_2 = \frac{\beta_2}{N}(X_{SI} + X_{II} + X_{CI} + X_{RI}) + \omega_2$.

More on inference technology

Consider a data set Y, consisting of observations of m variables $y_i(t_j), i = 1, \ldots, m$ at n points in time $t_j, j = 1, \ldots, n$. These data reflect indirectly on the true state of the system via the joint probability distribution of $y_i(t_j)$ on the system's state $x(t_j)$ at time t_j. In particular, specifying an observation model is equivalent to postulating the probability distribution $f(y \mid x, t, \theta)$ of observing the data $y(t)$ given the state of the system x at time t. In this context, θ represents a vector of unknown parameters to be estimated.

For likelihood-based inference, we calculate the likelihood that a chosen parameter set θ explains the complete data (within the confines of the process and observation models). This likelihood function $\mathcal{L}(\theta)$ is a product of conditional likelihoods, $\mathcal{L}_{t_j}(\theta)$, calculated at each time t_j for all n data points in time. In practice, it is more convenient to calculate the natural logarithm of the likelihood function, $\log \mathcal{L}(\theta)$, which involves taking sum of the conditional log-likelihoods.

$$\begin{aligned}
\mathcal{L}(\theta) &= f(y(t_1), y(t_2), \ldots, y(t_n)|\theta) \\
&= \prod_{j=1}^{n} f_\theta(y(t_j)|y(t_{j-1}), y(t_{j-2}), \ldots, y(t_1)) \\
&= \prod_{j=1}^{n} \mathcal{L}_{t_j}(\theta). \\
\log \mathcal{L}(\theta) &= \sum_{j=1}^{n} \log \mathcal{L}_{t_j}(\theta).
\end{aligned}$$

The Markov property of the model allows one to calculate the conditional likelihood $\mathcal{L}_{t_j}(\theta)$ sequentially starting from $t_j = t_1$. For each t_j, the likelihood function is:

$$\begin{aligned}
\mathcal{L}_{t_j}(\theta) &= f_\theta(y(t_j)|y(t_{1:j-1})) \\
&= \int\int \overbrace{f_\theta(y(t_j)|x(t_j))}^{\text{observation model}} \overbrace{f_\theta(x(t_j)|x(t_{j-1}))}^{\text{process model}} \overbrace{f_\theta(x(t_{j-1})|y(t_{1:j-1}))}^{\text{filtering distribution}} \,\mathrm{d}x(t_j)\,\mathrm{d}x(t_{j-1})
\end{aligned}$$

Furthermore, the calculation of the filtering distribution is simplified identifying a recursive relation by applying Bayes' Theorem.

$$\begin{aligned}
f_\theta(x(t_{j-1})|y(t_{1:j-1})) &= \frac{f_\theta(y(t_{j-1})|x(t_{j-1}))\, f_\theta(x(t_{j-1})|y(t_{1:j-2}))}{\int f_\theta(y(t_{j-1})|x(t_{j-1}))\, f_\theta(x(t_{j-1})|t(t_{1:j-2}))\,\mathrm{d}x(t_{j-1})} \\
&= \frac{\overbrace{f_\theta(y(t_{j-1})|x(t_{j-1}))}^{\text{observation model}} \overbrace{f_\theta(x(t_{j-1})|y(t_{1:j-2}))}^{\text{filtering dist.}}}{\underbrace{\mathcal{L}_{t_{j-1}}(\theta)}_{\text{cond. loglik}}}
\end{aligned}$$

The density functions $f_\theta(\cdot|\cdot)$ are calculated via Sequential Monte Carlo particle filtering method [1, 2].

Construction of log-likelihood profiles

Each log-likelihood profile is constructed on the basis of log-likelihoods calculated at various points in a two dimensional grid, where the two dimensions represent the two parameters that we are inferring. For Figs. 3, 4, 5, 9, S1, and S2, they are the short term interaction ($\phi = \xi$), and the long term interaction (χ). For Fig. S3, the parameters varied were reporting rate (ρ) and long term interaction χ. The grids span between the values of 0 and 2 for ϕ and χ, and we sampled at intervals of 0.2. For, ρ, the grid spans between 0.1 and 1, and we sampled at intervals of 0.1. When the results warranted refining, additional calculations were performed at finer intervals. This was necessary for profiles of χ, and ρ since the peaks were much sharper. The points where these additional samples were taken can be read off directly from the graphs.

We estimate log-likelihoods at each of these sampled points in the grid. We performed at least 5 separate calculations for each sampled point, each using 30,000 particles. Likelihood values are averaged over these replicates, and log of this mean is reported as the log-likelihood estimate.

Profiles were then constructed for both of the parameters. This was done by fixing the parameter being profiled at different sampled values and maximizing over the other parameter. A smooth line was then fitted through the values at the sampled points. We used local polynomial regression fitting (`loess`) provided in `R` [3] with span of 0.75 for profiles of ϕ and 0.5 for χ and ρ. The 95% confidence interval is taken to be $\chi_1^2(0.95)/2 \approx 1.92$ log-likelihood units below the maximum — univariate confidence limits using the χ^2 distribution.

The log-likelihood surfaces shown in Fig. 6 follows the same process, except here the parameter χ is fixed and parameters δ and $\phi = \xi$ are varied. A smooth 2-dimensional surface is fitted using approximate Nadaraya Watson kernel smoother, implemented in `R`-package `fields`. The function `smooth.2d` is used with bandwidth of 0.11 and resolution of 257 by 257. The 95% confidence interval is taken to be $\chi_2^2(0.95)/2 \approx 3$ log-likelihood units below the maximum — bivariate confidence limits using the χ^2 distribution. While the choice of the bandwidth and resolution can somewhat affect the precise boundary of the 95% confidence interval, the qualitative picture remains unaltered.

Log-likelihood surface shown in Fig. 8 is a two-dimensional representation of the results used for Fig. S3 without any smoothing. The 95% confidence interval is taken to be $\chi_2^2(0.95)/2 \approx 3$ log-likelihood units below the maximum — bivariate confidence limits using the χ^2 distribution.

For Fig. S-4, the profiles were constructed over χ, with $\phi = \xi = 0.6$.

Changing epidemiology

All of the our analysis was based on epidemiology of a disease with R_0 of 2.7. Here, we look at a disease with higher R_0 ($\beta = 130$, and $R_0 = 5$) under scenario III — partial and temporary cross-immunity, and delayed but permanent enhancement. We compare the inference results between the two diseases in Fig. S1. The inferences for a disease with higher R_0 are more precise. This is especially noticeable for the short term interaction. This is to be expected — the dynamical impact of interaction on a disease with larger R_0 is larger, and consequently much easier to infer.

More on under-reporting

We conducted two separate sets of experiments to address problems associated with under-reporting. In the first, we asked if we are still able to infer interactions if the data presented were under-reported. To mimic this, we scaled a previously generated data by 50%, attempted to infer both short- and long-term interaction, with prior knowledge of 50% reporting rate. The profiles are shown in Fig. S2.

In the second, we asked if we are able to infer interactions from an under-reported data, when under-reporting rate is not known *a priori.* In the paper, we presented a log-likelihood surface, for reporting rate ρ and long-term interaction χ (Fig. 8). In Fig. S3, we present log-likelihood profiles and the simulated data corresponding to the surface.

Estimating initial conditions

Our default estimation experiments assumed that initial conditions — the fractions of the host population in each compartment, X_{SS} through X_{RR} — are known *a priori.* In practice, initial conditions would have to be estimated along with the interaction parameters. Here, we relax the assumption that initial conditions are known, and attempt to infer simultaneously the interaction parameters and initial conditions. We take the two data sets from scenario III previously examined in Fig. 5 and attempt to infer the long-term interaction parameter, χ.

We proceed as follows:

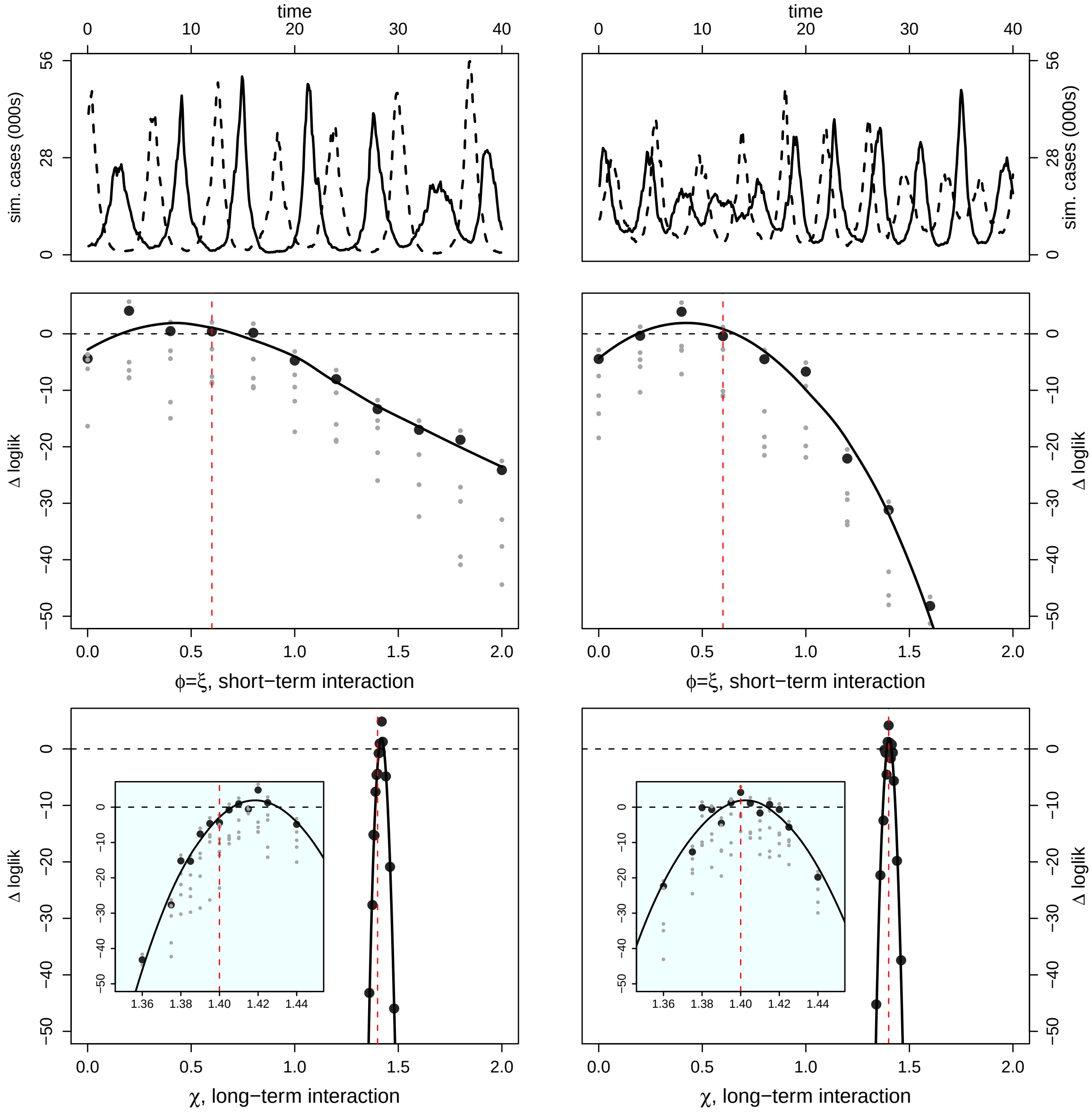

Figure S1. Inference under scenario III, with different epidemiology. Inference is carried out for two separate data sets in scenario III – [Left] with $\beta = 70$ ($R_0 \approx 2.7$) and [Right] with $\beta = 130$ ($R_0 \approx 5$). [Top] Simulated case-data for the two infections are plotted in solid and dashed lines. Log-likelihood profiles for parameters describing the short ($\phi = \xi$) [Middle] and the long term (χ) [Bottom] interactions. In the insets, we show close-ups of the profiles near the peaks. Plotted Δloglik are relative difference in the raw log-likelihood from the reference point set at $\Delta\text{loglik} = 0$, indicated by the horizontal dashed line. $\Delta\text{loglik} = 0$ represents the 95% confidence interval — parameter values corresponding to a positive Δloglik are within the confidence bound. The gray dots indicate the repeated likelihood estimates (5 replicate SMC calculations for each profile point, $30,000$ particles in each SMC calculation). The profiles are created by fitting a smooth line through the log of the mean likelihoods (shown in black dots). The vertical red dashed line is plotted at the actual parameter value used to generate the simulated case-data. Parameters not shown in the graph are taken from Table 1.

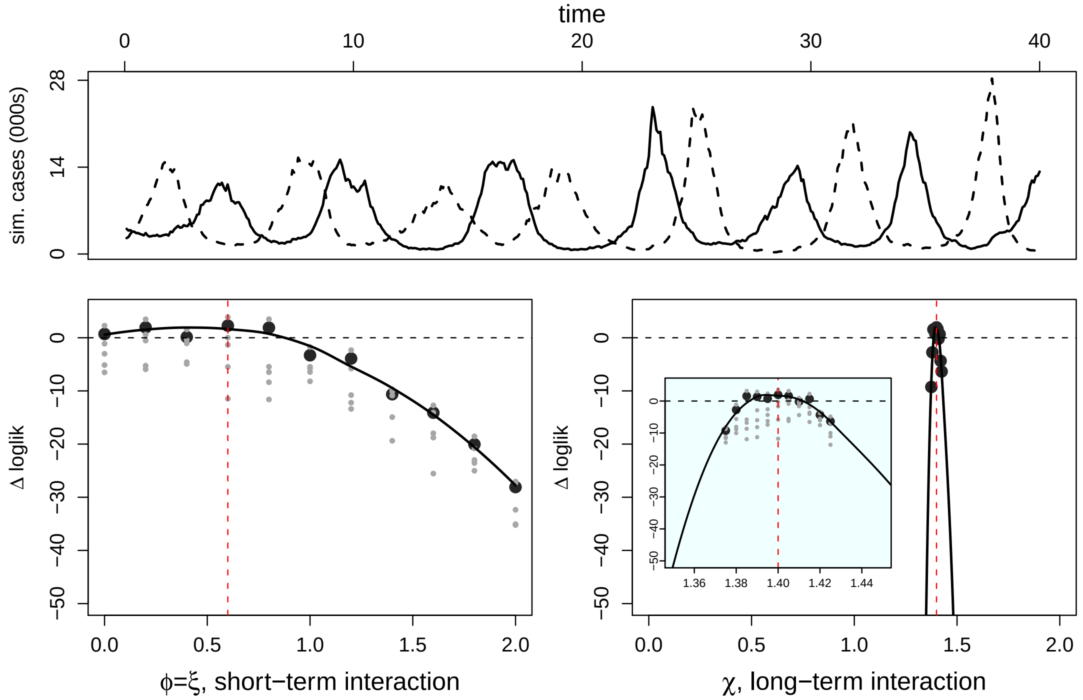

Figure S2. Inference under scenario III with under-reporting. [Top] Simulated case-data for the two infections are plotted in solid and dashed lines. These are the same data set used to make Fig. 5 [Right], but with 50% reporting rate. Log-likelihood profiles for parameters describing the short ($\phi = \xi$) [Bottom-Left] and the long term (χ) [Bottom-Right] interactions. In the inset, we show close-up of the profile near the peak. Plotted Δloglik are relative difference in the raw log-likelihood from the reference point set at Δloglik $= 0$, indicated by the horizontal dashed line. Δloglik $= 0$ represents the 95% confidence interval — parameter values corresponding to a positive Δloglik are within the confidence bound. The gray dots indicate the repeated likelihood estimates (5 replicate SMC calculations for each profile point, $30,000$ particles in each SMC calculation). The profiles are created by fitting a smooth line through the log of the mean likelihoods (shown in black dots). The vertical red dashed line is plotted at the actual parameter value used to generate the simulated case-data. Parameters not shown in the graph are taken from Table 1.

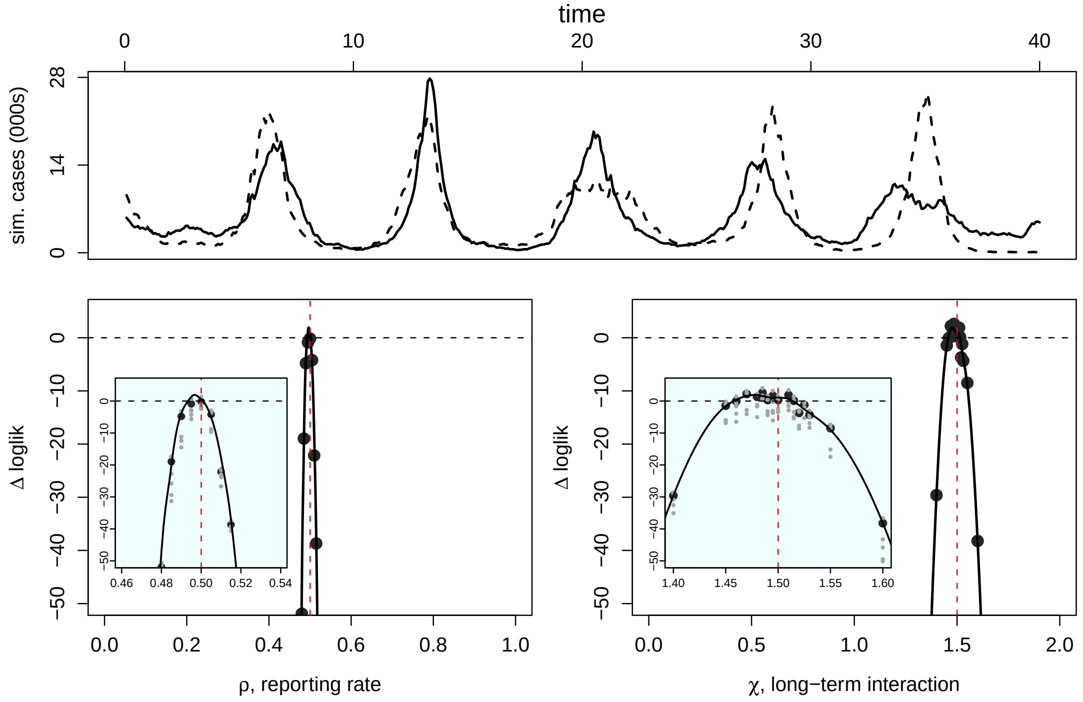

Figure S3. Simultaneous inference of under-reporting and long-term interaction. [Top] Simulated case-data for the two infections are plotted in solid and dashed lines. Log-likelihood profiles for reporting rate (ρ) [Bottom-Left] and the long term interaction (χ) [Bottom-Right]. In the inset, we show close-up of the profiles near the peak. Plotted Δloglik are relative difference in the raw log-likelihood from the reference point set at $\Delta\text{loglik} = 0$, indicated by the horizontal dashed line. $\Delta\text{loglik} = 0$ represents the 95% confidence interval — parameter values corresponding to a positive Δloglik are within the confidence bound. The gray dots indicate the repeated likelihood estimates (5 replicate SMC calculations for each profile point, $30,000$ particles in each SMC calculation). The profiles are created by fitting a smooth line through the log of the mean likelihoods (shown in black dots). The vertical red dashed line is plotted at the actual parameter value used to generate the simulated case-data. Here, short term interaction is absent ($\phi = \xi = 1$), and other parameters not shown in the graph are taken from Table 1.

1. For each profile point, we simulate the deterministic skeleton of the model to obtain the asymptotic range of each state variable.

2. We generate 100 guesses of initial conditions within this range.

3. For each guess, we attempt to maximize the fit of the model to the first 5 years of the data by varying only the initial conditions. We quantify the fit using the synthetic log-likelihood [4] based upon several summary statistics (probes). The probes used include means, variances, and marginal data distributions ignoring the time ordering. The optimization was done with a Nelder-Mead algorithm and the `probe.match` function in the `R` package [3] `pomp` [5].

4. We then select the initial condition estimate with the highest synthetic likelihood and proceed as described previously.

In Fig. S4, we present two profiles for the long term interaction, χ. In blue are the profiles based on the true initial conditions, while in black we present profiles with estimated initial conditions. They are plotted in the same log-likelihood scale to enable comparison. As expected, since more parameters have been estimated, the profiles in black are broader. Crucially, the long-term interaction remains well-identified, *i.e.* χ is not fundamentally confounded with initial conditions.

References

1. Ionides EL, Breto C, King AA (2007) Modeling Disease Dynamics: Cholera as a Case Study, Wiley, Hoboken NJ, chapter 8. pp. 13—140.

2. Arulampalam MS, Maskell S, Gordon N, Clapp T (2002) A tutorial on particle filters for online nonlinear/non-gaussian bayesian tracking. IEEE transactions on signal processing 50: 174—188.

3. R Development Core Team (2009) R: A Language and Environment for Statistical Computing. R Foundation for Statistical Computing, Vienna, Austria. URL `http://www.R-project.org`. ISBN 3-900051-07-0.

4. Wood S (2010) Statistical inference for noisy nonlinear ecological dynamic systems. Nature 466: 1102–1104.

5. King AA, Ionides EL, Breto CM, Ellner S, Kendall B (2010). pomp: Statistical inference for partially observed markov processes (R package). http://pomp.r.forge.r-project.org.

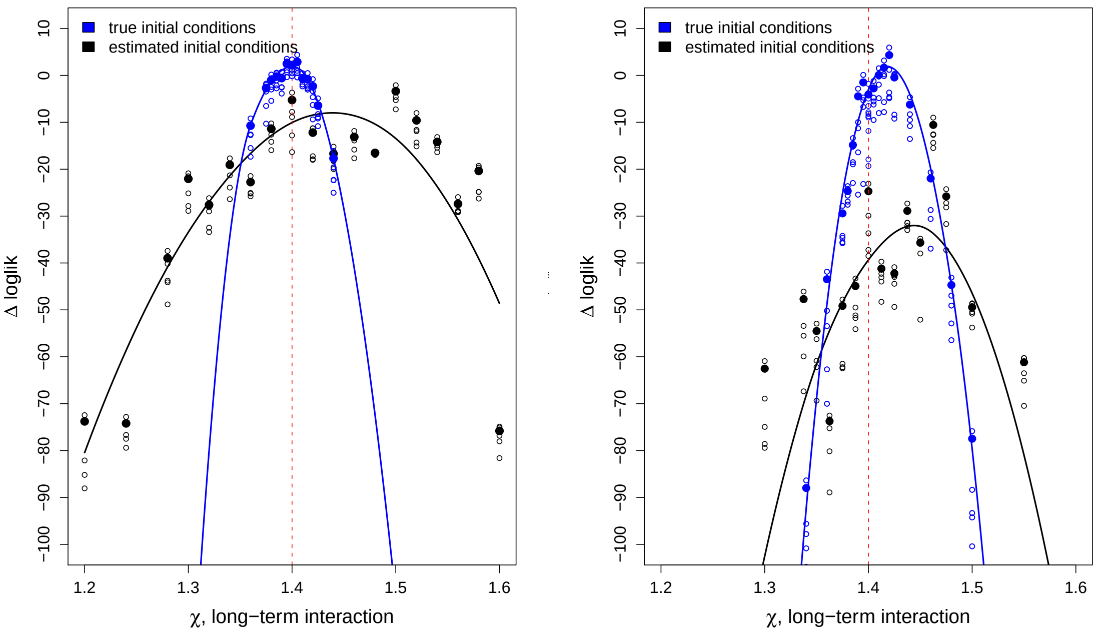

Figure S4. Inference of long-term interaction with estimated initial conditions. Log-likelihood profiles for the long-term interaction (χ), with true initial conditions [in blue] and estimated initial conditions [in black]. [Left] corresponds to the dataset used in Fig. 5[Left], and [Right] corresponds to the dataset used in Fig. 5[Right]. The empty dots indicate the repeated likelihood estimates (5 replicate SMC calculations for each profile point, $30,000$ particles in each SMC calculation). The parabolas have been fit to the logarithm of the mean likelihoods (shown in filled dots) and are intended only to guide the eye. The vertical red dashed line is plotted at the true parameter value used to generate the simulated case data. Here, short term interaction is ($\phi = \xi = 0.6$), and other parameters not shown in the graph are taken from Table 1.
